# Tumor-restricted activation of Vγ9Vδ2 T cells via bispecific Evobodies: a novel strategy for safe and potent immunotherapy in ovarian cancer

**DOI:** 10.3389/fimmu.2025.1628501

**Published:** 2025-07-18

**Authors:** Hans-Heinrich Oberg, Malte Deseke, Steffen Krohn, Dorothee Winterberg, Matthias Peipp, Dirk Bauerschlag, Klaus-Peter Künkele, Daniela Wesch, Christoph Baumann

**Affiliations:** ^1^ Institute of Immunology, University Hospital Schleswig-Holstein, Kiel, Germany; ^2^ Evobright GmbH, Vienna, Austria; ^3^ Division of Antibody-Based Immunotherapy, University Hospital Schleswig-Holstein and Christian-Albrechts University, Kiel, Germany; ^4^ Polyclinic and Clinic of Gynecology, University Center, Jena, Germany

**Keywords:** Vγ9Vδ2 T cells, FOLR1, evobody, ovarian cancer, immunotherapy, BTN3A

## Abstract

**Introduction:**

Vγ9Vδ2 T cells have been clinically evaluated—both *in vivo* and *ex vivo*—for their efficacy against solid tumors over several decades. Although recent therapeutic approaches have renewed hope, significant and reproducible benefits for patients with solid tumors remains to be demonstrated.

**Material & Methods:**

We have developed bispecific biologics in an IgG-extended format that bind both to Folate Receptor alpha (FOLR1), which is highly expressed in the majority of ovarian cancers, and to the activating butyrophilin (BTN)3A. By reducing the affinity of the BTN3A agonist and leveraging the increased avidity of the tetravalent, bispecific antibody, activation of BTN3A is restricted to FOLR1-positive tumors, thereby avoiding off-target activation of non-tumor cells.

**Results:**

Using RTCA co-culture assays with Vγ9Vδ2 T cells and tumor cell lines, we identified “Evobodies” that exhibit a strong therapeutic window and high potency against FOLR1-positive cells, while sparing healthy, FOLR1-negative tissue. Moreover, the lead molecule demonstrates high efficacy in a human autologous, patient-derived *ex vivo* tumor tissue model at unaltered/physiological effector-to-target (E:T) ratios. Importantly, we show that our tumor-engaging molecules avoid premature immune cell activation, degranulation, and cytokine release in the absence of FOLR1-positive tumor cells. They likely establish a cytokine gradient from the tumor site, harnessing the full potential of the natural local anti-infection response to target cancer cells.

**Discussion:**

Thus, Evobodies represent a novel, first-in class of biologics for solid tumor treatment and are well-suited for further clinical development.

## Introduction

Immunotherapies using bispecific antibodies or CAR T cell therapies aimed at achieving durable and effective responses in solid tumors such as epithelial ovarian cancer (EOC) are under investigation (1). EOC is the most lethal gynecological malignancy in women. The first-line therapy of EOC is surgical resection followed by platinum-based chemotherapy, with response rates of approximately 80% (2). However, recurrence is a major challenge and is often associated with resistance to platinum-based chemotherapeutic standard regimen (3). To improve patient survival, different therapeutic strategies are being explored.

Folate receptor alpha (FRα or FOLR1) is a tumor-associated cell surface antigen expressed in ~80% of EOC cases and is nearly absent in normal tissues (4). In a randomized phase II trial, the anti-FOLR1 monoclonal antibody Farletuzumab, combined with standard chemotherapy, failed to improve progression-free survival in patients who were platinum-sensitive during their first relapse and had low levels of the tumor marker cancer antigen (CA) 125 (5). A phase I expansion study of Mirvetuximab soravtansine-gynx (MIRV), an antibody-drug conjugate targeting FOLR1, showed promising results and (6, 7) was subsequently approved in 2024. MIRV has been recognized as a novel therapy with demonstrated efficacy and a favorable risk-benefit profile in platinum-resistant ovarian cancer (8). A systematic meta-analysis described the efficacy and safety profile of the FOLR1-targeting antibody therapy in EOC and fallopian tube cancer patients revealed substantial heterogeneity and variability in treatment efficacy depending on disease status (9, 10). To further improve treatment efficacy and tolerability, novel alternative strategies such as immune cell targeting of FOLR1-positive ovarian cancers, are needed to further improve treatment efficacy and tolerability.

Current immunotherapy approaches using CD3 T cell activating bispecific antibodies in solid tumors have been fueled by successes in hematologic malignancies (11, 12). Notably, Tarlatamab, a CD3 bispecific antibody, was approved for a subset of lung cancer patients (13). However, a large patient population still remains with high medical needs. Moreover, target -mediated side effects such as cytokine release syndrome (CRS) caused by tumor distant activation of immune cell cytokine release in the peripheral blood (14) and the activation of immunosuppressive Tregs (15) can induce effects the counteract efficacy and tolerability.

Vγ9Vδ2 T cells are the major subpopulation of γδ T cells and are important versatile effector cells of the mammalian immune system sharing functions of the adaptive and the innate immune system (16, 17). These include cytotoxic activity against infected cells, antigen presentation to CD4 and CD8-positive T cells, rapid expansion in response to local infection, homing to infected tissues, and secretion of proinflammatory cytokines to promote pathogen clearance (18). Even though less in abundance compared to CD8 T cells, Vγ9Vδ2 T cells have been found to infiltrate solid tumors in patterns distinct from CD8 T cells, and their presence has been associated with improved patient survival in several cancer indications (19). Since virtually every tumor harbors a microbiome in a subpopulation of tumor cells (20–22), it can be speculated that the mode of clearance of infected tissue by Vγ9Vδ2 T cells is active in solid tumors. Consequently, harnessing the activity of Vγ9Vδ2 T cells by *ex vivo* or *in vivo* activation for cancer therapy has gained interest in the past decades. However, clinical studies have not been sufficiently convincing to translate these hopes into clinical efficacy (23).

Butyrophilin (BTN)3A (also known as CD277) is a membrane protein that acts as a sensitive infection sensor by detecting phosphoantigens produced by metabolically active bacteria (24, 25). It is ubiquitously expressed in human tissues and signals infection by undergoing conformational changes to the TCR of Vγ9Vδ2 T cells allowing them to recognize infected cells (26). BTN3A has gained interest as a drug target, and agonistic antibodies have been developed to activate BTN3A at the cell surface (27, 28).

Interestingly, in tumor cell - Vγ9Vδ2 T cell co-culture experiments, pre-incubation of a BTN3A agonist with target cells — but less so with immune effector cells — led to tumor cell elimination (24) This observation supports the idea that efficient killing of tumor cells by Vγ9Vδ2 T cells can occur when BTN3A is activated specifically on the target cell (24).

Here, we introduce the Evobody, a bispecific antibody that selectively activates BTN3A on FOLR1-positive tumor cells, thereby mimicking a localized bacterial infection and triggering a BTN3A-dependent immune response. Evobodies circumvent activation of ubiquitously expressed BTN3A on any cell of the body but rather activate tumor cell surface exposed BTN3A in strong dependency of the presence of the tumor anchor. We show that this tumor -restricted “kill me” signal results in selective cytokine secretion and establishes a localized immune gradient. Moreover, Evobodies induce effector cell proliferation and avoid premature effector cell degranulation and self-elimination initiated by untargeted BTN3A activation with the BTN3A agonist Bromohydrin pyrophosphate (BrHPP) (29). Finally, Evobodies induce potent tumor cell elimination and Vγ9Vδ2 T cell proliferation at very low E:T rations in *ex vivo* freshly isolated patient tumors with endogenous TILs and avoid immune exhaustion by circumventing the activation of CD4 Tregs or upregulation of T cell immunoreceptor with Ig and ITIM domains (TIGIT).

## Results

### Evobodies are first-in-class bispecific therapeutic antibodies that mimic an intracellular infection selectively at the tumor site

**Figure 1:**
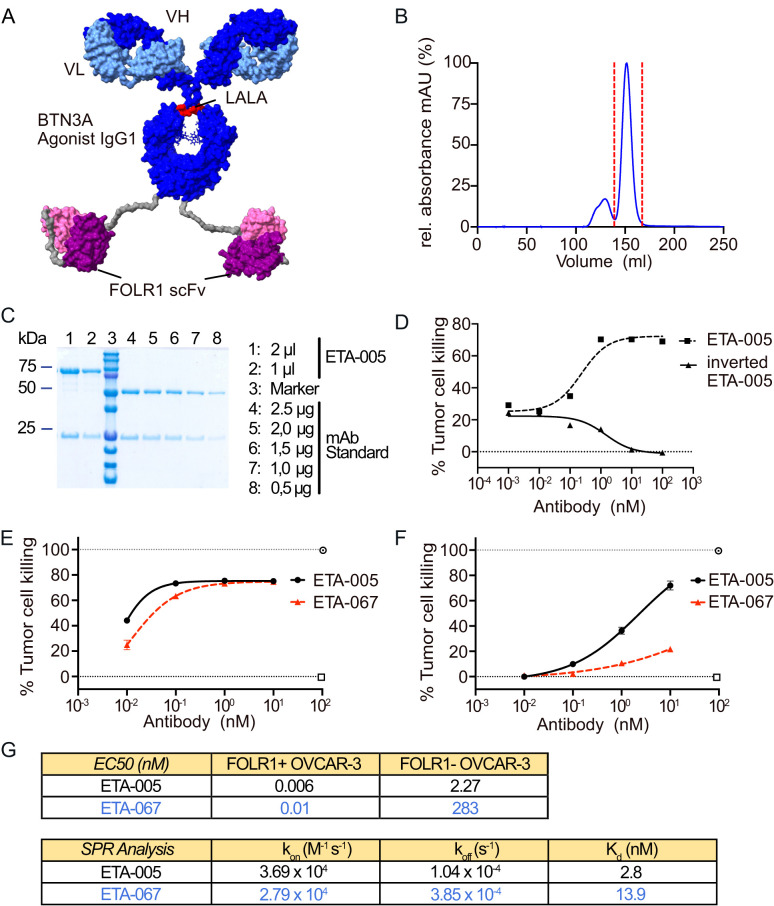
Generation and Optimization of bispecific Evobodies. **(A)** Homology model of the estimated structure of an Evobody. Dark blue: heavy chain of IgG1 agonist binding to BTN3A engineered with an Fc LALA mutation (L234A/L235A) to abrogate Fcγ receptor binding and effector functions, light blue: corresponding light chain, purple: scFvs binding to FOLR1, grey: linker sequences. **(B)** Chromatogram of the size exclusion chromatography (SEC) of recombinantly expressed Evobody. The red dashed line indicates the peak fractions that were collected and contained the correctly assembled Evobody. **(C)** SDS-PAGE of purified Evobody after SEC with two different volumes being loaded. For comparison, a mAb standard was loaded at decreasing amounts. 10% gel with reducing conditions. **(D)** Dose response curve of ETA-005 and the inverted format ETA-005 determined via an RTCA cytotoxicity assay with expanded Vγ9Vδ2 T cell lines from a healthy donor co-cultured in triplicates with OVCAR-3 cells. **(E, F)** Dose response curves for ETA-005 (before germline reset) and ETA-067 (after germline reset) determined via an RTCA cytotoxicity assay with expanded Vγ9Vδ2 T cells from a healthy donor co-cultured in triplicates either with OVCAR-3 wild-type cells **(E)** or with FOLR1 knock-out cells **(F)**. **(G)** Comparison of ETA-005 and ETA-067 regarding their EC50 values from cytotoxicity assays with FOLR1-positive WT OVCAR-3 cells and FOLR1-negative cells (Top table) and their K_d_-values as from surface plasmon resonance experiments to determine their affinity to BTN3A (bottom table).

For the design of a bispecific antibody targeting BTN3A and a tumor-restricted antigen to activate Vγ9Vδ2 T cells at the tumor site, two main requirements had to be considered: first, activation of BTN3A with antibodies requires bivalent binding (30) and second, tumor anchor binding requires sufficient affinity to the tumor-restricted protein to ensure tumor dependent binding and activation of the effector target (31). Thus, we chose a symmetric, bispecific format with a bivalent monoclonal antibody paired with two single chain variable fragments (Mab-ScFv format) (32) and integrated publicly available V-regions from an agonistic BTN3A antibody (24, 33) (**Figure 1A**). We selected the tumor-specific antigen FOLR1, which is highly expressed in ovarian carcinoma (4, 34), a tumor indication with a high medical need for new therapeutic approaches (2). With its high and stable expression, FOLR1 supports the local recruitment and concentration of the bispecific antibody in proximity to the co-expressed but less abundant BTNA3 (**Supplementary Figure 1A**). Moreover, V-region sequences for the FOLR1 binding scFvs of Farletuzumab have been published previously (35, 36). After the expression of antibody constructs in CHO cells and subsequent purification via affinity chromatography, a single major peak was detected in the subsequent size exclusion chromatography (SEC) step (**Figure 1B**). This suggests that the expressed antibody sequences pair well and do not have the tendency to form aggregates due to mispairing. The major peak was collected for further analysis.

Analytical SDS-PAGE under reducing and non-reducing conditions confirmed the expected sizes of the heavy chain fused with a scFv (75 kDa) and the light chain (25 kDa), as well as the high purity of the antibody solution (**Figure 1C**, **Supplementary Figure 1B**). Taken together, we concluded that the antibody construct was well expressed and shows a high degree of purity as required for further *in vitro* tests with primary immune cells.

To test whether the localization of the antigen-binding domains — either in the IgG variable region or in the attached scFvs — affects functionality, molecules with both possible orientations of the V-regions were generated (**Supplementary Figure 2A**). These constructs were then tested for their ability to induce a cytotoxic response by expanded Vγ9Vδ2 T cells against OVCAR-3 cells using a Real-Time Cell Analysis (RTCA) assay. The molecule featuring a BTN3A-binding IgG and tumor anchor-specific scFvs induced tumor cell killing, whereas the inverted format—with scFvs specific for BTN3A—did not (**Figure 1D**). This indicates that binding of the bispecific molecules to BTN3A requires the corresponding V-regions to be located within the IgG-derived portion of the molecule.

Since the BTN3A agonistic antibody alone can bind BTN3A with high affinity and induce Vγ9Vδ2 T cell activation without tumorrestriction (24), we performed a germline reset of the BTN3A -specific V-regions. By decreasing the affinity for BTN3A, activation was confined to cells where the high-affinity binding of the scFvs to FOLR1 enabled increased avidity and proximity of the BTN3A-binding domains to BTN3A. The tumor-killing response induced by the resulting Evobodies was assessed using the RTCA cytotoxicity assay. To determine the specificity for FOLR1-positive cells, we generated a FOLR1 knockout (KO) in OVCAR-3 cells via CRISPR/Cas9 (**Supplementary Figure 2B**). Comparing the cytotoxic responses of each candidate molecule against OVCAR-3 wild-type (WT) and FOLR1 KO cells allowed us to determine the respective therapeutic window was determined. The screening culminated in the Evobody ETA-067 as the version with the largest difference in EC50 between WT and FOLR1 KO cells (**Figures 1E, F**). Additionally, variant ETA-062, which differed from ETA-067 by a single amino acid at position 31 in the light chain, exhibited similar activity in the RTCA assay and was also included as a lead molecule (**Supplementary Figure 2C**). Interestingly, for the pre-germline-reset parental antibody ETA-005, the reactivity against FOLR1 KO cells was already reduced in comparison to WT cells indicating that the molecular design itself already leads to an increased specificity towards cells expressing the tumor anchor FOLR1. We hypothesize that the addition of scFvs may induce conformational changes — unexamined here — that reduce BTN3A affinity. Nevertheless, the mutations in ETA-067 lead to a strong decrease in the response to FOLR1 KO cells in comparison to ETA-005 as reflected by the EC50 whereas the response against FOLR1-positive WT cells remained at similar levels (**Figure 1G**). Affinity measurements via surface plasmon resonance (SPR) of ETA-005 and ETA-067 towards immobilized BTN3A further corroborated this observation as the K_d_ of ETA-067 was decreased by about four-fold in comparison to ETA-005. Therefore, we chose for ETA-067 as a lead molecule for further characterization due to its efficacy on FOLR1-positive cells paired with reduced tumor antigen independent activation.

### Evobodies induce the release of cytokines and proliferation of Vγ9Vδ2 T cells specifically at the tumor site and avoid premature degranulation

**Figure 2:**
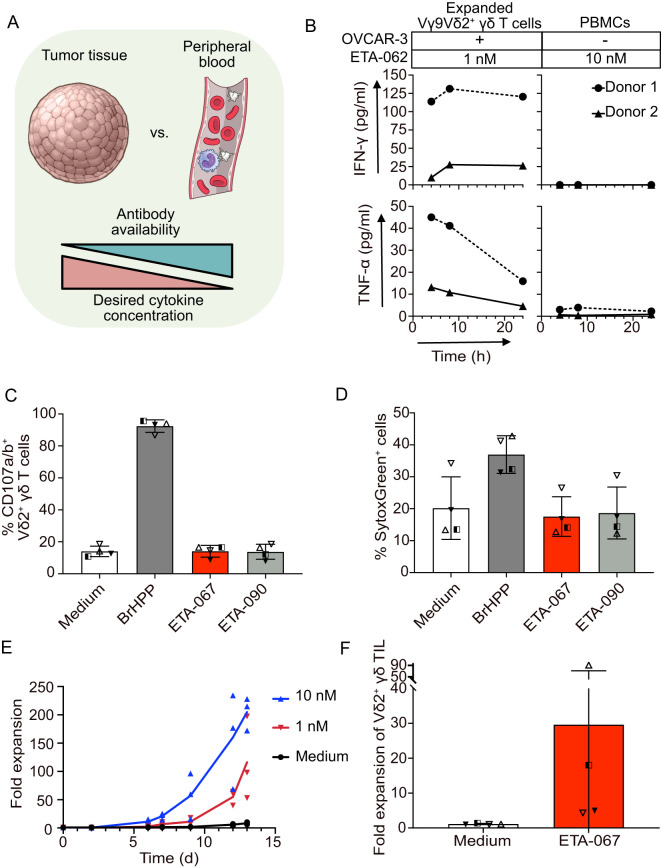
Evobodies can induce the release of activating cytokines and proliferation of Vγ9Vδ2 T cells specifically at the tumor site. **(A)** Schematic representation of the comparison of tumor tissue and peripheral blood with regard to estimated Evobody availability and desired cytokine concentration. Created with Illustrations from NIAID NIH BIOART Source. **(B)** Expanded Vγ9Vδ2 T cells from healthy donors were co-cultured with OVCAR-3 cells for up to 24 h while donor-matched PBMCs were cultured for the same time period without OVCAR-3 cells. Evobody ETA-062 was added at concentrations of 1 nM to expanded Vγ9Vδ2 T cells and 10 nM to PBMCs. Supernatants were taken after 4, 8 and 24 h and the concentration of IFN-γ and TNF-α was determined via ELISA. Every line represents one donor (n=2 donors). (C/D) Vγ9Vδ2 T cell lines plus rIL-2 from healthy donors (n=4) were cultured in medium or with 300 nM BrHPP or 1 nM of the indicated Evobodies in the presence of anti-human CD107a mAb. **(C)** The percentage of CD107-positive Vδ2 T cells and **(D)** of dead cells stained by SytoxGreen was measured after 4 h by flow cytometry. **(E)** PBMCs from healthy donors were co-incubated with OVCAR-3 cells for up to 13 days in the presence of 50 IU/ml rIL-2. Evobody ETA-067 was added at 10 or 1 nM concentration while the culture with rIL-2 alone (Medium) served as negative control. The Vδ2 γδ T cells were counted via FACS staining and the acquisition of a specific volume (n=3 donors). **(F)** *Ex vivo* isolated Vδ2 TIL within tumor tissue were cultured in medium or stimulated with ETA-067 together with repetitive addition of IL-2 (every 3 days). After 10 to 14 days, the absolute number of expanded Vδ2 TIL in medium or ETA-067 stimulated cultures were determined and x-fold Vδ2 TIL expansion of the four donors in four different experiments was calculated.

The release of cytokines is a major hallmark of immune cell activation and a key requirement for the induction of a broader immune response by bispecific antibodies in the tumor (37, 38). At the same time, the systemic release of cytokines caused by off-target activation of immune cells causes severe side effects and should be avoided (14). Therefore, it is desirable to induce a gradient of activating and inflammatory cytokines that peaks at the tumor site and is lowest in the periphery, mimicking the immune environment during a primary infection (37, 39) (**Figure 2A**).

To verify whether Evobodies can selectively induce cytokine release at the tumor site while sparing peripheral blood cells, we designed a cytokine release assay. A realistic co-culture model, however, should reflect the differences in the activation status of Vγ9Vδ2 T cells at different locations. Under physiological conditions, the cytokine gradient between tumor and periphery is driven by pre-activated, tumor-infiltrating T cells in the tumor and resting immune cells in the peripheral blood. Hence, we used pre-expanded Vγ9Vδ2 T cells from healthy donors co-cultured with OVCAR-3 cells as a simplified model of the tumor and PBMCs without target cells to model the peripheral blood. To avoid elevated cytokine release simply due to the higher number of Vγ9Vδ2 T cells in pre-expanded cultures, the number of pre-expanded Vγ9Vδ2 T cells and resting Vγ9Vδ2 T cells in the corresponding PBMC samples were matched according to the Vδ2 cell frequencies determined previously.

Another important consideration for a realistic comparison between peripheral blood and tumor was the availability of the bispecific antibody in the patient. As the antibody is administered intravenously, its concentration would be highest in the bloodstream and lower in the tumor tissue, forming a gradient that is inversely related to the desired cytokine release. Therefore, to incorporate the differences in antibody concentrations between peripheral blood and tumor in the assay, 10 nM Evobody was added to the PBMC cultures, while 1 nM was added to the pre-expanded Vγ9Vδ2 T cell co-cultures. Finally, the levels of the activating cytokines IFN-γ and TNF-α in the supernatant were quantified using ELISA.

In an experiment with cells from two healthy donors over a time course of 24 hours, Evobody ETA-062 did not elicit any release of IFN-γ and triggered only low levels of TNF-α when added to PBMCs alone (**Figure 2B**). In contrast, pre-expanded Vγ9Vδ2 T cells co-cultured with OVCAR-3 cells secreted both IFN-γ and TNF-α upon addition of ETA-062. This effect was observed despite the low number of Vγ9Vδ2 T cells in the co-culture and the 10x lower Evobody concentration. This suggests that Evobodies can likely cause a cytokine gradient between tumor tissue and the blood stream despite low abundance of Vγ9Vδ2 TILs and lower concentration of the Evobody in comparison to peripheral blood.

Next, we aimed to investigate whether a conventional CD3 bispecific T cell engager format added to PBMCs without OVCAR-3 tumor cells being present would induce the release of IFN-γ and TNF-α. Therefore, we generated the CD3 bispecific molecule ETA-100, based on the previously described therapeutic antibody Farletuzumab (40), (**Supplementary Figures 3A–D**). A subsequent cytokine release assay with PBMCs showed that, although ETA-067 does not induce IFN-γ or TNF-α release, the CD3 bispecific molecule caused tumor anchor-independent production of both cytokines after 24 hours of culture (**Supplementary Figures 3E, F**). This result is in line with previous reports on the induction of CRS in patients treated with CD3 bispecifics (14, 41). In contrast, Evobodies restrict cytokine production to the tumor, mimicking the localized immune activation seen in infections. These data suggest that Evobodies may circumvent CRS -related safety concerns in patients and support the development of a proinflammatory immune gradient originating from the tumor.

To further corroborate that Evobodies do not induce premature cytotoxic responses, we performed a degranulation assay. Briefly, pre-expanded Vγ9Vδ2 T cells from healthy donors were incubated without tumor cells but in the presence of Evobody ETA-067. As a positive control, the cells were treated with the phosphoantigen BrHPP, which induces conformational changes in BTN3A in all cells, whether immune or tumor. An Evobody (termed ETA-090) where the BTN3A agonist CDRs have been replaced with an irrelevant sequence, specific for hen-egg lysozyme, and medium without additional molecules were used as the negative controls. The surface presence of CD107a and CD107b, serving as indicators of degranulation in immune cells, was used to measure the activation of the expanded Vγ9Vδ2 T cells. In line with the results from the cytotoxicity experiments with FOLR1 KO tumor cells described earlier, ETA-067 did not induce the premature, tumor anchor-independent degranulation in pre-activated Vγ9Vδ2T cells (**Figure 2C**). In addition, no increased killing of other bystander immune cells was observed with ETA-067 as was assessed by the number of dead cells in the cultures compared with the medium control (**Figure 2D**). However, when BTN3A was activated without restriction to the tumor cell surface —as in the case of BrHPP — an increased number of dead cells was detected. We therefore deduced that the Vγ9Vδ2 T cells began committing fratricide, as the conformational changes in BTN3A were also induced in the Vγ9Vδ2 effector cells themselves. This further highlights the importance of restricting BTN3A — and thereby Vγ9Vδ2 T cell — activation to the tumor site. Otherwise, immune effector cells that are relevant for the anti-tumor immune response and cells of surrounding healthy tissue might be targeted as well, thereby compromising the desired anti-tumor immune response. The results from the degranulation assay suggest that Evobodies avoid premature degranulation of effector cells while also sparing non-tumor cells from off-target Vγ9Vδ2 T cell-mediated killing.

Another critical aspect to address in the use of Vγ9Vδ2 T cells for anti-tumor immunotherapy is their typically low abundance within the tumor. It is therefore beneficial that Evobodies not only induce killing of target cells and tumor-restricted cytokine release but also promote the proliferation of Vγ9Vδ2 T cells to amplify their anti-tumor effects. To assess this, we performed an *in vitro* proliferation assay. PBMCs from healthy donors were cultured for up to 13 days with OVCAR-3 cells in the presence of either 1 nM or 10 nM of Evobody ETA-067 and IL-2 to support growth. Cell numbers were subsequently determined by FACS analysis. Over the course of the experiment, the number of Vγ9Vδ2 T cells increased ~200-fold with 10 nM ETA-067 and ~100-fold with 1 nM ETA-067, whereas cultures without Evobody showed no proliferation (**Figure 2E**). This suggests that the activation of Vγ9Vδ2 T cells by Evobodies also specifically induces their proliferation. Interestingly, other Vγ9Vδ2-negative T cells also expanded in the presence of ETA-067, though to a lesser extent (**Supplementary Figure 3G**). The extent to which this effect might be due to the alleviated inhibitory effect of BTN3A on αβ T cells (28) or other factors such as cytokines produced by Vγ9Vδ2 T cells (18) or allogeneic reactivity remains to be investigated.

To investigate whether proliferation can also be observed in patient-derived tumor-infiltrating cells, we stimulated *ex vivo* –isolated Vδ2 TILs within tumor tissue with either medium or ETA-067, along with the repeated addition of IL-2 (every 3 days). After 10 to 14 days, we observed varying levels of Vδ2 TIL expansion (**Figure 2F**). The patient-dependent variation of the Vδ2 T cell expansion correlated with the individually varying, physiological amount of autologous ovarian tumor cells and other immunosuppressive cells in the four different experiments with four different ovarian tumor patients. To assess whether *ex vivo* Vδ2 TILs from these patients exhibited an exhausted phenotype, we analyzed the expression of programmed cell death protein (PD)-1 and T cell immunoreceptor with Ig and ITIM domains (TIGIT) of the Vδ2 TIL. *Ex vivo* isolated Vδ2 TILs did not express PD-1 or TIGIT (data not shown). These results demonstrate that ETA-067, together with repeated IL-2 administration, can induce expansion of Vδ2 TILs at the tumor site.

### Evobodies mediate Vγ9Vδ2 T cell-cytotoxicity against autologous ovarian tumor cells

**Figure 3:**
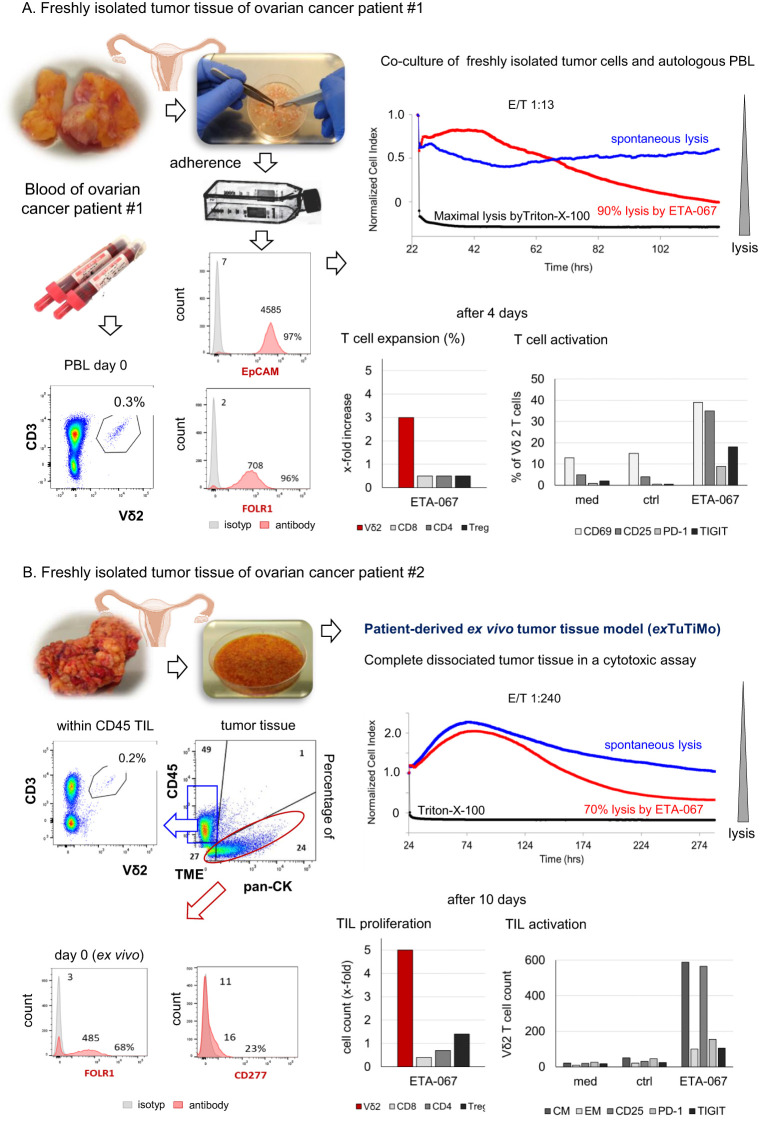
Two different patient-derived *ex vivo* models. **(A, B)** Freshly isolated tumor tissue from ovarian cancer patients was minced and dissociated for two different models. **(A)** Dissociated tumor tissue was cultured in complete medium overnight. After 24 h, adherent cells were collected and stained for EpCAM and FOLR1 expression. Autologous PBL were isolated from the blood and stained for CD3-positive Vδ2 T cells. Adherent tumor cells and autologous PBL were cultured in the presence of 1 nM ETA-067 and 50 IU/mL rIL-2 with an E/T ratio of 1:13 over 4 days to determine Vδ2 T cells cytotoxicity, expansion and the percentage of activation (CD69, CD25) and inhibitory check point molecules (PD-1 and TIGIT) on Vδ2 T cells and other T cells (CD4, CD8, Treg). **(B)** For the novel patient-derived *ex vivo* tumor tissue model (exTuTiMo) complete freshly isolated, dissociated tumor tissue including an immunosuppressive tumor-microenvironment was plated and stimulated with or 1 nM control construct ETA-090 (spontaneous lysis) or 1 nM ETA-067 together with 50 IU/mL rIL-2 in complete medium with an unchanged physiological effector/target ratio of 1:240. Cytotoxicity was measured over the whole time and flow cytometric analysis of the absolute cell number (cell count > proliferation) and expression of activation (CD25) and inhibitory markers (PD-1 and TIGIT) markers as well as of central and effector memory cells (CM, EM) was determined initially and at an endpoint on Vδ2, CD4, CD8 or Treg TILs (10 days after culture).

Enhancement of immune cell cytotoxicity against tumor cells is one important feature of bispecific antibodies. To analyze whether ETA-067 induces enhanced cytotoxicity of Vγ9Vδ2 T cells within peripheral blood lymphocytes (PBL) or Vγ9Vδ2 tumor-infiltrating lymphocytes (TIL) from ovarian cancer patients, two different models were used (**Figures 3A, B**). For both models, freshly isolated tumor tissue was minced and dissociated directly after surgery. In the model in which we examined the Vγ9Vδ2 T cell cytotoxicity within PBL, we co-cultured freshly isolated PBL with freshly isolated, autologous tumor cells (**Figure 3A**). In addition, Vγ9Vδ2 T cell cytotoxicity within TIL was determined with our novel established *ex vivo* patient-derived tumor tissue model (*ex*TuTiMo), in which Evobodies selectively activate cells within freshly isolated human tumor tissue, including an immunosuppressive tumor microenvironment (**Figure 3B**).

In the co-culture of adherent EpCAM-positive ovarian tumor cells, which highly expressed FOLR1, with autologous PBL from patient #1, stimulation with ETA-067 and rIL-2 induced enhanced lysis (90%) of the tumor cells (**Figure 3A**). Four days after stimulation with ETA-067, Vγ9Vδ2 T cells in PBL were expanded and activated, as demonstrated by increased percentages of CD25- and CD69-positive Vδ2 T cells. The percentage of PD-1 and TIGIT-expressing cells was only slightly increased in Vδ2 T cells (**Figure 3A**).

For patient #2, we first analyzed the composition of cells in the freshly dissociated tumor tissue *ex vivo*. The tissue consisted of 24% pan-CK–positive tumor cells, which expressed FOLR1 and CD277 (BTN3A), 49% CD45-positive leukocytes, and 27% tumor-surrounding cells (including stromal and epithelial cells) (**Figure 3B**). Both the percentage and absolute number of Vδ2 TIL among tumor-infiltrating lymphocytes were very low. After *ex vivo* stimulation of the dissociated tumor tissue with ETA-067 and IL-2, we observed 70% lysis of ovarian tumor cells by ETA-067–stimulated Vδ2 TIL. In an endpoint flow cytometry analysis of the cultured tissue after 10 days, we found an increased absolute number of CD25-expressing Vδ2 TIL with a central memory phenotype, which had proliferated (~5-fold expansion) and showed only slight expression of PD-1 or TIGIT (**Figure 3B**). Taken together, these results demonstrate that ETA-067 can stimulate either PBL or TIL within tumor tissue to exert enhanced cytotoxic activity against autologous ovarian tumor cells.

## Discussion

BTN3A is an integral plasma membrane protein that functions in inside -out signaling as an omnipresent infection sensor, expressed on every cell of our body (18, 42, 43)and is activated on cells carrying intracellular metabolically active bacteria (44, 45). In fact, very small amounts of the pathogenic phosphoantigen ligand 1-hydroxy-2-methyl-2-buten-4-yl 4-diphosphate (HDMAPP) are sufficient to trigger this “trap wire” and signal the presence of intracellular pathogens to the extracellular environment (18, 46). Likewise, a very low number of BTN3A molecules on the plasma membrane is enough to receive and translate the infection signal to activate proximal Vγ9Vδ2 T cells (18). The subsequent reaction is highly potent and comprises a versatile and complex response — including pro-inflammatory cytokine release (47), antigen cross-presentation of pathogenic and tumor-antigens (48), cross-activation of other immune cells including CD4 and CD8 T cells (28), massive expansion of Vγ9Vδ2 T cells (49), and ultimately, elimination of the infected BTN3A - activated target cell (24).

It is an important question whether Vγ9Vδ2 T cells and their anti-infection biology play a natural role in anti-tumor responses. In fact, approximately 1% of cells in every tumor are infected with intracellular bacteria (20), and it is tempting to speculate that the overall beneficial role of Vγ9Vδ2 TILs in patient survival may (19) be attributed to their intrinsic, natural activity of Vγ9Vδ2 T cells against bacterial infection in tumors. These cells patrol tissues and tumors and, although present at low frequency, are — like BTN3A — nearly omnipresent. This contrasts with CD8 T cells, whose infiltration into tumors varies widely depending on mutation load and other factors (19). Hence, based on the distribution and potency of Vγ9Vδ2 T cells, harnessing and multiplying their natural anti-tumor response is a highly attractive concept to battle solid tumors. Understandably, Vγ9Vδ2 T cells have attracted the interest of many researchers and drug developers for over three decades (23). Beyond several variations to administer phosphoantigens directly to patients or to systemically increase their natural, intracellular levels through amino-bisphosphonate administration (18), a key breakthrough was the identification and characterization of a BTN3A agonistic antibody (24, 30). Notably, if this agonistic antibody was pre-incubated with target cells prior to a co-culture with Vγ9Vδ2 T cells, it induced efficient tumor cell killing. However, pre-incubation of the agonist with Vγ9Vδ2 T cells did not result to tumor cell killing in subsequent co-culture, indicating (and following the infection biology) that the BTN3A infection signal must be set on the cell to be eliminated. Thus, it seems reasonable to conclude that activating BTN3A at the tumor site would have higher efficacy than direct targeting of effector cells (50, 51).

Consequently, we generated bispecific, tetravalent molecules with a bivalent, low-affinity agonistic binder to BTN3A to limit BTN3A engagement in the absence of the second, tumor -specific epitope. Additionally, the bispecific antibody (bsAB) binds the tumor-associated antigen FOLR1 with high affinity. In co-culture assays, we demonstrated that a >1000x higher potency can be achieved when FOLR1 is present on the cell surface. Thus, BTN3A can be selectively activated on tumor cells, avoiding undesired binding and activation in non-target tissues.

Other bispecific molecules, such as CD3 T cell engagers that bridge a tumor-associated antigen with an effector cell target (12), are designed for a similar purpose: to activate effector cells and eliminate tumors. For instance, Ubamatamab is a CD3xMuc16 bispecific antibody currently being developed in relapsed ovarian cancer patients (52). However, key differences exist between activating a signal at the target cell (as with Evobodies) and activating the effector cell (via CD3 engagement). Immune cell-engaging biologics such as CD3 -activating antibodies bind directly to immune cells. In consequence, those CD3 T cells may become exhausted due to the absence of a co-stimulatory signal (53, 54). Additionally, activation of all CD3-positive T cells can lead to widespread cytokine release, not only contributing to severe side effects such as CRS but also preventing the establishment of a tumor-specific cytokine gradient selectively from the tumor (14, 55). Moreover, premature degranulation and effector cell exhaustion (14, 56) reveal well known obstacles in establishing a tumor directed, long lasting immune response. Evobodies in contrast activate Vγ9Vδ2 T cells from the tumor and induce the establishment of a local immune response including cytokine release and effector cell proliferation — key prerequisites for overcoming the highly immunosuppressive tumor microenvironment of solid tumors (39). Thus, the unique property of Evobodies to activate Vγ9Vδ2 T cells by selectively triggering the BTN3A infection alarm on FOLR1 -positive tumor cells distinguishes them from pan-BTN3A activating approaches (27, 28) as well as from virtually all bispecific concepts, where the administered therapeutic antibody is – by itself – building an non-physiological, molecular bridge from the TAA/FOLR1 to an immune effector cell.

Translational models in immuno-oncology remain a bottleneck in drug development with diverse limitations (57): commonly used *in vitro* assays are short-term, involve very high effector-to-target (E:T) ratios, and poorly reflect the complexity of patient tumor biology (18) In contrast, *in vivo* models often require transgenic mice, adoptive transfers, or surrogate molecules to simulate the biology of a given therapeutic concept. This limitation also applies here, as neither BTN3A nor Vγ9Vδ2 T cells are conserved in rodents (58). Increasingly accepted by drug developers, *ex vivo* modeling using human tumor tissue and immune cells is considered the “closest-to-patient” approach for generating predictive efficacy (18). However, patient sample availability, tissue quality, and assay setup are key factors for maintaining translational validity, and thus, we present a 10-day long term assay named patient-derived *ex vivo* tumor tissue model (exTuTiMo), which measures efficacy in technical triplicates using primary ovarian tumor samples with an unaltered, patient -derived cellular composition of tumor cells and tumor-infiltrating lymphocytes (TILs). Indeed, the ultimate assessment of efficacy remains to be addressed in clinical trials in cancer patients. However, this model presents a very high-level approximation: The experiment we show has an E:T ratio of 1:240, representing the usually low levels of Vγ9Vδ2 effector T cells found in patient tumors. Nevertheless, Evobodies demonstrated strong efficacy against patient tumor cells, induced robust effector cell proliferation, and avoided upregulation of exhaustion markers — suggesting that long -term efficacy may be achievable in patients with high medical need.

In summary, Evobodies are novel, first -in -class bispecific molecules that harness the natural anti-infection biology by eliciting the BTN3A infection signal specifically at the tumor site, thereby initiating an anti-tumor immune response from within the tumor. itself. Highly sophisticated preclinical *ex vivo* models using patient tumors suggest that Evobodies promote Vγ9Vδ2 T cell proliferation, prevent immune cell exhaustion, and eliminate tumors at low initial E:T ratios. Therefore, they may offer promising long-term efficacy in tumor antigen-positive patients. 

## Materials and methods

### Expression and purification of the antibody derivatives

Antibodies were produced in CHO-S cells by transient transfection, as previously described (59). A two-step purification process was used. In the first step Capture Select IgG-CH1-XL affinity matrix (Thermo Fisher Scientific, Bremen, Germany) was used for affinity chromatography. In the second step, size-exclusion chromatography (ÄKTA pure, GE Healthcare, Chicago, IL, USA/Cytiva, Uppsala, Sweden) was performed to remove traces of multimers or aggregates, as previously described (59).

### SDS-PAGE analysis

The indicated volume or weight of each purified recombinant protein was loaded onto 12% Tris–acrylamide gels under reducing conditions, or onto 4–15% precast polyacrylamide gels (Mini-PROTEAN^®^ TGX™, Bio-Rad, Hercules, CA, USA) under non-reducing conditions, and stained using Coomassie Brilliant Blue staining solution (Carl Roth GmbH, Karlsruhe, Germany), as previously described (59).

### Antibody structure modeling

The protein structure model of the Evobody was generated using MODELLER and Chimera X software (60, 61).

### Surface plasmon resonance

Surface plasmon resonance (SPR) experiments were performed at NBS-C, Vienna, using a Biacore™ 3000 instrument (Serial No.: 31-1122395-3531). Certified -grade CM5 optical sensor chips were utilized for all measurements. Recombinant BTN3A samples were immobilized onto the surface of a Biacore CM5 optical sensor chip via covalent EDC/NHS coupling, following the Biacore amine coupling kit protocol. Throughout all experiments, Biacore HBS-EP buffer was used as the running buffer. Instrument handling and operation were conducted in accordance with the protocols outlined in the Biacore^®^ 3000 Instrument Handbook. Kinetic analysis of the binding sensorgrams was performed using the BIA evaluation 4.1 software. Appropriate application protocols and mathematical curve-fitting models were applied as specified in the BIA evaluation Software Handbook.

### Patient cohort

Informed written consent was obtained from all donors in accordance with the Declaration of Helsinki, and the research was approved by the relevant institutional review boards (the ethics committee of the Medical Faculty of the CAU Kiel and the ethics committee of the Austrian Red Cross, Vienna). Leukocyte concentrates from healthy adult blood donors were obtained from the Department of Transfusion Medicine of the UKSH in Kiel (ethics committee code number: 405/10) and the blood donation center of the Austrian Red Cross (ethics committee code number: 20210506_01). EDTA blood and tumor tissue from ovarian cancer patients were obtained from the Department of Gynecology and Obstetrics at the University Hospital Schleswig-Holstein (UKSH) in Kiel, Germany (code number: D 445/18).

### Establishment of Vγ9Vδ2 T cell lines

Peripheral blood lymphocytes (PBL) were isolated from leukocyte concentrates or EDTA blood of healthy or ovarian cancer patient donors using Ficoll-Hypaque™ PLUS (Cytiva) density gradient centrifugation. A total of 1 x 10^6^ PBMCs were stimulated with 2.5 μM of the aminobisphosphonate (n-BP) zoledronate (Novartis, Basel, Switzerland) in complete medium.

The complete culture medium for primary immune cell culture or assay setup was composed of RPMI 1640 supplemented with 2 mM L-glutamine, 25 mM HEPES, 100 U/mL penicillin, 100 µg/mL streptomycin, and 10% FCS. All cell culture procedures and assay incubation steps were performed at 37°C in a humidified atmosphere with 5% CO2. Activation of PBL with n-BP and IL-2 induced selective activation and proliferation of Vγ9Vδ2 T cells. Recombinant IL-2 (Novartis) was added every 2 days at a concentration of 50 IU/mL for 14 days, as initially stimulated γδ T cells produced only low levels of IL-2. After 14 days, the short-term activated γδ T cell lines were stained with the following antibodies: AF700-labeled anti-CD3 clone SK7 (BioLegend, San Diego, CA, USA); AF488-labeled anti-Vγ9 clone 7A5 (62) flow cytometry to determine purity. γδ T cells with a purity of >95% were used for functional assays.

### Cancer cell line and culture media

The ovarian cancer cell line OVCAR-3 was obtained from ATCC and cultured in complete medium (OVAR-3 medium). For continuous subculture, the cells were split every 2–3 days using 0.25% Trypsin/EDTA for detachment.

### Generation of a FOLR1-negative cell line

An OVCAR-3 cell line with a knock-out (KO) for FOLR1 was generated using CRISPR/Cas9. The respective gRNA sequence used was CAAGGAAAAGCCAGGCCCCG. A pool of FOLR1-negative and -positive cells was obtained from Synthego, and single -cell clones were generated via limited dilution. The resulting clones were tested for FOLR1 expression via flow cytometry using a PE-labeled anti-FOLR1 antibody (clone LK26, BioLegend), and a KO cell line was established from a clone that was completely negative for FOLR1.

### Cytotoxicity assay (Real-Time Cell Analysis)

The γδ T cell cytotoxicity against adherent OVCAR-3 cells (WT or FOLR1 KO) was analyzed in triplicate using a Real-Time Cell Analysis system (RTCA; Agilent Technologies, Inc., Santa Clara, CA, USA). A total of 10,000 tumor cells per well were seeded in an E-plate with goldelectrodes at the bottom, in complete medium, to monitor cell impedance every 5 minutes for up to 24 hours. After the tumor cells reached a linear growth phase, γδ T cell lines supplemented with 12.5 IU/mL rIL-2 were added to the 96-well micro-E-plate. The cells were cultured in medium alone or stimulated with different concentrations of ETA-005, ETA-062, or ETA-067. A final concentration of 1% Triton X-100 per well was added to several wells as a positive control for tumor cell death. Raw data files were exported from RTCA Software 2.0 to Microsoft Excel and/or GraphPad Prism to calculate the cytotoxic potential of γδ T cells toward tumor cells. The mean of the Triton X-100 samples was calculated and defined as 100% lysis at various time points. The lysis ratio for each sample relative to spontaneous tumor cell death was calculated, normalized to the maximum lysis induced by Triton X-100, and corrected by subtracting the medium control. Dose –response curve fitting was performed in PRISM with non-linear fit, dose response, variable slope, top constraint model analysis.

### Cytokine release assay

For co-culture of expanded Vγ9Vδ2 T cells with OVCAR-3 cells, 20,000 tumor cells were seeded per well in a flat-bottom 96-well plate in OVCAR-3 medium and cultured overnight. Wells intended for culturing PBMCs alone were left blank. The next day, frozen pre-expanded Vγ9Vδ2 T cells were thawed and cultured overnight in complete medium supplemented with 20% FCS and 25 IU/ml of rhIL-2 (Peprotech, Cranbury, NJ, USA) for recovery. On day 2, autologous PBMC samples donor-matched to the pre-expanded Vγ9Vδ2 T cells were also thawed and cultured for 1h in complete medium with 20% FCS. Subsequently, 2 x 10^5^ PBMCs were added to the blank wells in complete medium with 10 nM Evobody ETA-062. Evobody ETA-062 differs from ETA-067 by a single amino acid and showed identical behavior in cytotoxicity assays. Therefore, ETA-062 was used in place of ETA-067 at the time of experimentation for practical reasons, which did not interfere with assay performance. To ensure equal absolute numbers of Vγ9Vδ2 T cells between PBMC and pre-expanded samples of the same donor, the pre-expanded Vγ9Vδ2 T cells were diluted in complete medium accordingly. Frequencies of Vγ9Vδ2 T cells in PBMC and expanded samples were determined by flow cytometry to calculate the required dilution. The diluted pre-expanded Vγ9Vδ2 T cells were then added to the pre-seeded OVCAR-3 cells with the Evobody ETA-062 at a concentration of 1 nM. Each combination of cell types and Evobody was pipetted in triplicates. Supernatants were collected from all samples after 4, 8 and 24 h respectively and centrifuged at 1200 rpm for 5 minutes. The quantification of IFN-γ and TNF-α in the supernatants was performed by sandwich ELISA using the ELISA MAX™ Deluxe Set Human IFN-γ and the ELISA MAX™ Deluxe Set Human TNF-α (both BioLegend) following the procedures outlined by the manufacturer. The ELISA was performed in uncoated Nunc™ MaxiSorp™ ELISA Plates (BioLegend). In total, 50 µl of each sample was diluted 1:2 with Assay diluent A provided in the ELISA Kits prior to the addition to the ELISA Plate. After finishing the procedure, the absorbance at 450 and 570 nm was measured using the i3x microplate reader (Molecular Devices, Silicon Valley, CA, USA), and data were analyzed using GraphPad Prism version 10.1.1.

### CD107a-degranulation assay

γδ T cell lines (E:T ratio: 12.5:1), supplemented with 12.5 U/mL rIL-2, were cultured in complete medium or stimulated with 1 nM ETA-067, 1 nM ETA-090, or 300 nM BrHPP for 24 hours. For the CD107aassay, 10 µL FITC-labeled anti-human CD107a mAb clone H4A3 (50 µg/mL, BioLegend) was added directly, while 3 µM monensin was added 1 h after co-culturing the cells. After an additional 3 h, Vδ2 γδ T cells were stained with APC-Vio770-labeled anti-Vδ2 mAb clone REA771 (Miltenyi Biotec) and dead cells with SytoxGreen and analyzed by flow cytometry.

### Proliferation assay

OVCAR-3 cells were seeded at a density of 20,000 cells per well in flat -bottom, culture-treated 96-well microtiter plates (Thermo Fisher Scientific) and cultured overnight. The next day, frozen PBMCs from healthy donors were thawed and cultured for 1 h in recovery medium (complete medium supplemented with 20% FCS). After the OVCAR-3 medium was removed, 50,000 PBMCs (E:T ratio: 2.5:1) were added per well along with either 1 nM or 10 nM of Evobody ETA-067, or medium alone. The medium was supplemented with 50 IU/mL rhIL-2 (Peprotech). Each condition (Evobody concentrations and medium control) was pipetted in triplicate to account for technical variation. Every second day, rhIL-2 was added at a concentration of 50 IU/ml IL-2. To determine the absolute number of Vδ2 γδ T cells, the respective samples were harvested either at day 0 or in increments of 2 days for a total of 13 days. The samples were treated with Human TruStain Fc Block (BioLegend) and subsequently stained for 20 minutes in FACS buffer (PBS + 1% FCS + 2 mM EDTA) using PE-labeled anti-Vδ2 antibody clone REA771 (Miltenyi Biotec) and APC-labeled anti-CD3 antibody clone OKT3 (BioLegend). Sytox Green (Thermo Fisher Scientific) was used according to the manufacturer’s instructions for Live/Dead discrimination.

A total of 100 µL from each sample was acquired using a CytoFLEX flow cytometer (Beckman Coulter, Krefeld, Germany), and data analysis was performed with FCS Express software version 7.16.0046.

### Chromium release assay

T cell-mediated cytotoxicity by bispecific CD3 T cell engaging antibodies was measured using a chromium release assay as previously described (63). Tumor cells were labeled with radioactive ^51^Cr and incubated with isolated T cells from healthy donor blood (Pan T cell; Miltenyi Biotec) at an effector - to - target (E:T) ratio of 10:1 for 18 h. The percentage of lysis was calculated from counts per minute (cpm) as follows: % lysis = (experimental cpm × basal cpm)/(maximal cpm × basal cpm) × 100.

### Different patient-derived *ex vivo* models

For both models, tumors from advanced ovarian cancer patients removed during surgery were dissected by pathologists at the UKSH. These freshly isolated tumor tissues were minced and treated with components A, H, and R of the Tumor Dissociation Kit (Miltenyi Biotec) for 1 h at 37°C in 5 mL PBS in a gentle MACS (Miltenyi Biotec).

### Co-culture of freshly isolated tumor cells and autologous PBL

Dissociated tumor tissue was cultured in complete medium under regular conditions overnight. Thereafter, adherent tumor cells were collected, washed and cultured alone or together with freshly isolated autologous PBL) at an E:T ratio of 1:13 in the presence of 1 nM ETA-067 and 50 IU/mL rIL-2 for 4 days. Before culturing, the percentage of CD3-positive Vγ9Vδ2 T cells within PBL and the expression of FOLR1 on overnight cultured epithelial cell adhesion molecule (EpCAM)-positive tumor cells was determined as described in the flow cytometry section. Cytotoxic activity of ETA-067–stimulated Vγ9Vδ2 T cells in the PBL against autologous tumor cells was analyzed over 4 days by RTCA. Afterward, fold expansion of Vδ2 T cells and expression of activation and inhibitory check point molecules was determined as the frequency of positive cells.

### Patient-derived *ex vivo* tumor tissue model (exTuTiMo)

A novel, predictive, patient-derived *ex vivo* tumor tissue model (exTuTiMo) was used to evaluate cytotoxicity, proliferation, and the absolute cell count of activation and inhibitory markers in ETA-067–stimulated γδ TIL initially and at an endpoint analysis (EU patent number: 25 164 902.6; US patent number: 19/084,885). In this model, freshly dissociated tumor tissue, including the intact immuno suppressive tumor micro environment (TME), was seeded directly — without cell labeling or separation — into appropriate culture plates. The physiological effector-to-target (E:T) ratio of 1:240 was maintained. Cultures were stimulated with 1nM ETA-067 or the control construct ETA-090, alongside 50 IU/mL rIL-2 in complete medium. While cytotoxic activity was measured over the whole time, endpoint flow cytometric analysis of the absolute cell number was determined after 10 days of culture.

### Flow cytometry and absolute cell number analysis of tumor samples and PBL

In total, 1–2 x 10^6^ PBL from healthy donors or ovarian cancer patients, or dissociated tumor tissue, were stained using a multicolor flow cytometry approach. To determine the distribution and activation status of T cells within CD45-positive leukocyte populations, the color panel included the following mAbs: PerCP-labeled anti-CD45 clone 2D1, PE-Cy7-labeled anti-TCR pan γδ clone 11F2 (both BD Biosciences), AF700-labeled anti-CD3 clone SK7, BV510- labeled anti-CD4 clone OKT4, APC-Cy7-labeled anti-CD8 clone SK1 (all three from BioLegend), as well as PE-Cy5-labeled anti-CD127 and PE-labeled anti-CD25 mAb clone REA945 (Miltenyi Biotec) to determine Treg, FITC-labeled anti-Vδ2 mAb clone IMMU389 (Beckman Coulter), APC-labeled anti-CD69 mAb clone FN50 (BioLegend), BV711-labeled anti-TIGIT mAb clone 741182 (BD Biosciences), and BV785-labeled anti–PD-1 (CD279) mAb clone EH12.2H7 (BioLegend), and a BV421-labeled viability marker (Thermo Fisher Scientific).

To determine the expression of FOLR1 on EpCAM-expressing ovarian tumor cells, 1 x 10^5^ tumor cells were stained with APC-labeled anti-EpCAM clone HEA-125 (Miltenyi Biotec) and PE-labeled anti-FOLR1 mAb clone LK26 (BioLegend).

To distinguish, in the dissociated tumor tissue (DiTuTi), between CD45-positive leukocytes, tumor cells, and stromal and epithelial cells of the tumormicroenvironment, the following mAbs were used: PerCP-labeled anti-CD45 clone 2D1 (BD Biosciences), PE-Vio770-labeled anti-HER-2 clone 24D2, and APC-labeled anti-EpCAM clone HEA-125 or APC-labeled anti-CD277 clone BT3.1 (all from Miltenyi Biotec), BV421-labeled anti-CD90 clone 5E10 (BioLegend), PE-labeled anti-FOLR1 clone LK26 (BioLegend), and corresponding isotype controls (BD Biosciences or Miltenyi Biotec). Thereafter, DiTuTi was intracellularly stained with FITC-labeled anti-pan-cytokeratin (CK) mAb clone CK3-6H5 (Miltenyi Biotec) or an appropriate isotype control. For the intracellular staining, cells were washed with staining buffer, fixed and permeabilized with the Cytofix/Cytoperm kit (BD Biosciences) for 20 min, following the procedures outlined by the manufacturer. Thereafter, the cells were washed twice with Perm/Wash by centrifugation, stained with anti-panCK or isotype control mAb for 25 min, washed again twice, and measured.

All samples were analyzed on an LSR-Fortessa flow cytometer (BD Biosciences) using Diva 9 software.

For determination of the absolute viable cell number, Trucount Tubes (#340334 from BD Biosciences) were used, and adherent cells were dissociated and collected with 0.05% trypsin/0.02% EDTA.

## Data Availability

The original contributions presented in the study are included in the article/**Supplementary Material**. Further inquiries can be directed to the corresponding author.
