# Correction: Tumor-restricted activation of Vγ9Vδ2 T cells via bispecific Evobodies: a novel strategy for safe and potent immunotherapy in ovarian cancer

**DOI:** 10.3389/fimmu.2025.1672018

**Published:** 2025-09-12

**Authors:** Hans-Heinrich Oberg, Malte Deseke, Steffen Krohn, Dorothee Winterberg, Matthias Peipp, Dirk Bauerschlag, Klaus-Peter Künkele, Daniela Wesch, Christoph Baumann

**Affiliations:** ^1^ Institute of Immunology, University Hospital Schleswig-Holstein, Kiel, Germany; ^2^ Evobright GmbH, Vienna, Austria; ^3^ Division of Antibody-Based Immunotherapy, University Hospital Schleswig-Holstein and Christian-Albrechts University, Kiel, Germany; ^4^ Polyclinic and Clinic of Gynecology, University Center, Jena, Germany

**Keywords:** Vγ9Vδ2 T cells, FOLR1, evobody, ovarian cancer, immunotherapy, BTN3A

In the published article, there was a mistake in the **Materials and methods** section. In the paragraph, **Establishment of Vγ9Vδ2 T cell lines**, there was an erraneous concentration of 2.5mM:

“Peripheral blood lymphocytes (PBL) were isolated from leukocyte concentrates or EDTA blood of healthy or ovarian cancer patient donors using Ficoll-Hypaque™ PLUS (Cytiva) density gradient centrifugation. A total of 1 x 10^6^ PBMCs were stimulated with 2.5 mM of the aminobisphosphonate (n-BP) zoledronate (Novartis, Basel, Switzerland) in complete medium.

The complete culture medium for primary immune cell culture or assay setup was composed of RPMI 1640 supplemented with 2 mM L-glutamine, 25 mM HEPES, 100 U/mL penicillin, 100 µg/mL streptomycin, and 10% FCS. All cell culture procedures and assay incubation steps were performed at 37°C in a humidified atmosphere with 5% CO2. Activation of PBL with n-BP and IL-2 induced selective activation and proliferation of Vγ9Vδ2 T cells. Recombinant IL-2 (Novartis) was added every 2 days at a concentration of 50 IU/mL for 14 days, as initially stimulated γδT cells produced only low levels of IL-2. After 14 days, the short-term activated γδ T cell lines were stained with the following antibodies: AF700-labeled anti-CD3 clone SK7 (BioLegend, San Diego, CA, USA); AF488-labeled anti-Vγ9 clone 7A5 (62) flow cytometry to determine purity. γδ T cells with a purity of >95% were used for functional assays”.

A correction has been made to the section **Materials and methods**, **Establishment of Vγ9Vδ2 T cell lines:**

“Peripheral blood lymphocytes (PBL) were isolated from leukocyte concentrates or EDTA blood of healthy or ovarian cancer patient donors using Ficoll-Hypaque™ PLUS (Cytiva) density gradient centrifugation. A total of 1 x 10^6^ PBMCs were stimulated with 2.5 μM of the aminobisphosphonate (n-BP) zoledronate (Novartis, Basel, Switzerland) in complete medium.

The complete culture medium for primary immune cell culture or assay setup was composed of RPMI 1640 supplemented with 2 mM L-glutamine, 25 mM HEPES, 100 U/mL penicillin, 100 µg/mL streptomycin, and 10% FCS. All cell culture procedures and assay incubation steps were performed at 37°C in a humidified atmosphere with 5% CO2. Activation of PBL with n-BP and IL-2 induced selective activation and proliferation of Vγ9Vδ2 T cells. Recombinant IL-2 (Novartis) was added every 2 days at a concentration of 50 IU/mL for 14 days, as initially stimulated γδ T cells produced only low levels of IL-2. After 14 days, the short-term activated γδ T cell lines were stained with the following antibodies: AF700-labeled anti-CD3 clone SK7 (BioLegend, San Diego, CA, USA); AF488-labeled anti-Vγ9 clone 7A5 (62) flow cytometry to determine purity. γδ T cells with a purity of >95% were used for functional assays”.

“A correction was also made to the **Results** section, **Evobodies are first-in-class bispecific therapeutic antibodies that mimic an intracellular infection selectively at the tumor site:**

This sentence was altered in error: “Comparing the cytotoxic responses of each candidate molecule against OVCAR-3 wild-type (WT) and FOLR1 KO cells allowed us to determine the respective therapeutic windows. window was determined.”

A correction has been made to the sentence: “Comparing the cytotoxic responses of each candidate molecule against OVCAR-3 wild-type (WT) and FOLR1 KO cells allowed us to determine the respective therapeutic window was determined.”

Additionally, a final error was made in the **Results** section, **Evobodies mediate Vγ9Vδ2 T cellcytotoxicity against autologous ovarian tumor cells:**

Part of this sentence was made redundant: “Understandably, for more than three decades Vγ9Vδ2 T cells have attracted the interest of many researchers and drug developers for over three decades (23).”

This sentence has now been corrected: “Understandably, Vγ9Vδ2 T cells have attracted the interest of many researchers and drug developers for over three decades (23).”

The original version of this article has been updated.

