# Supplementary material for: Tumor-restricted activation of Vγ9Vδ2 T cells via bispecific Evobodies: a novel strategy for safe and potent immunotherapy in ovarian cancer
Source: Front Immunol. 2025 Jul 18;16:1628501. doi: 10.3389/fimmu.2025.1628501 (PMC12314882; doi:10.3389/fimmu.2025.1628501)
Supplement: Supplementary file 1 [file DataSheet1.docx]

***Supplementary Material***

**Supplementary Figures**

**
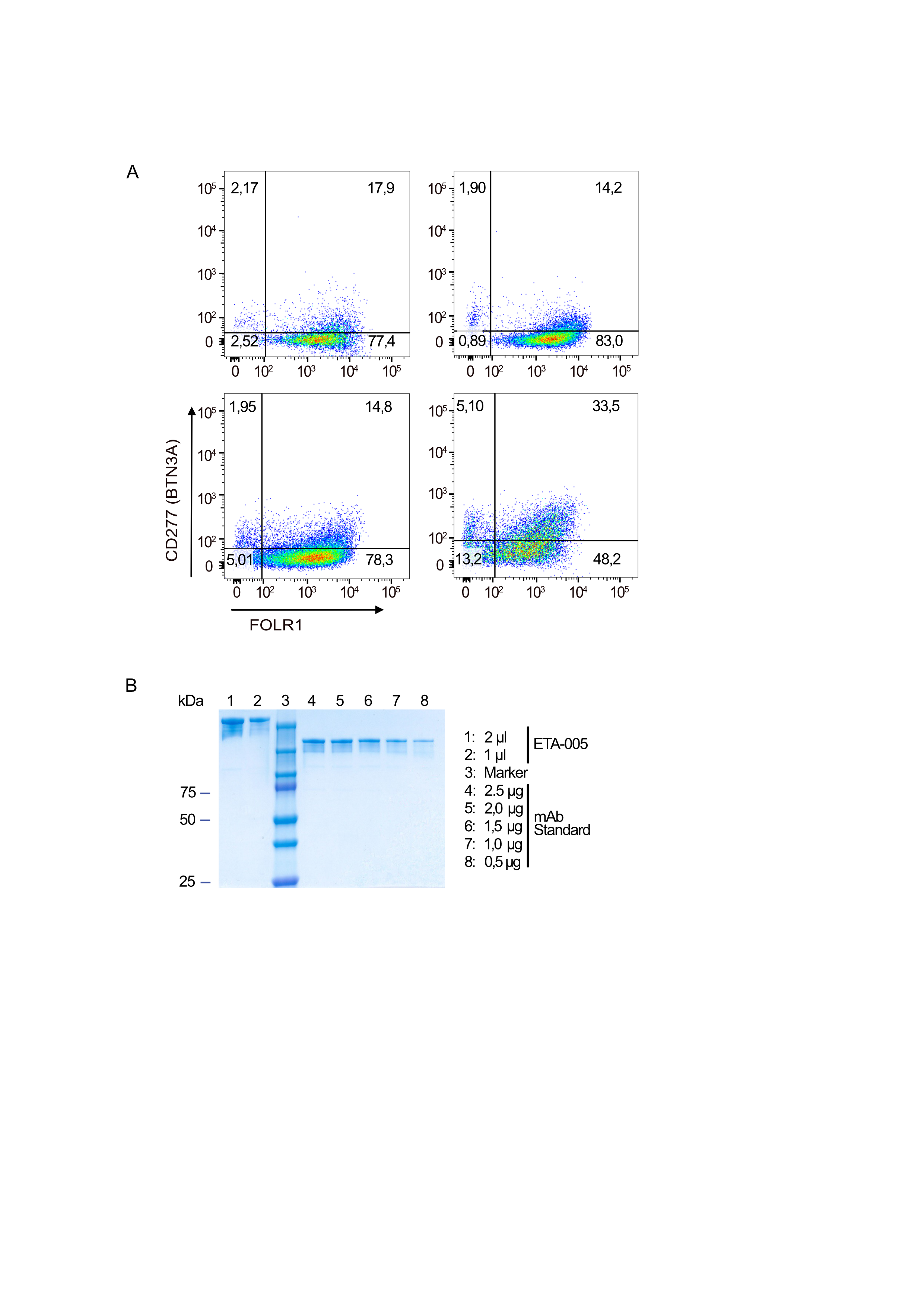
**

**Supplemental Figure 1: A:** Co-expression of CD277 and FOLR-1 on tumor cells of four different patients was analyzed by flow cytometry. Cells were gated on pan-cytokeratin-positive and CD45-negative cells within dissociated tumor tissue for CD277 and FOLR-1 surface expression. Each dot plot represents one donor. **B:** Non-reducing SDS-PAGE (4 – 15% gradient gel) of purified Evobody after SEC with two different volumes being loaded. For comparison, a mAb standard was loaded at decreasing amounts.


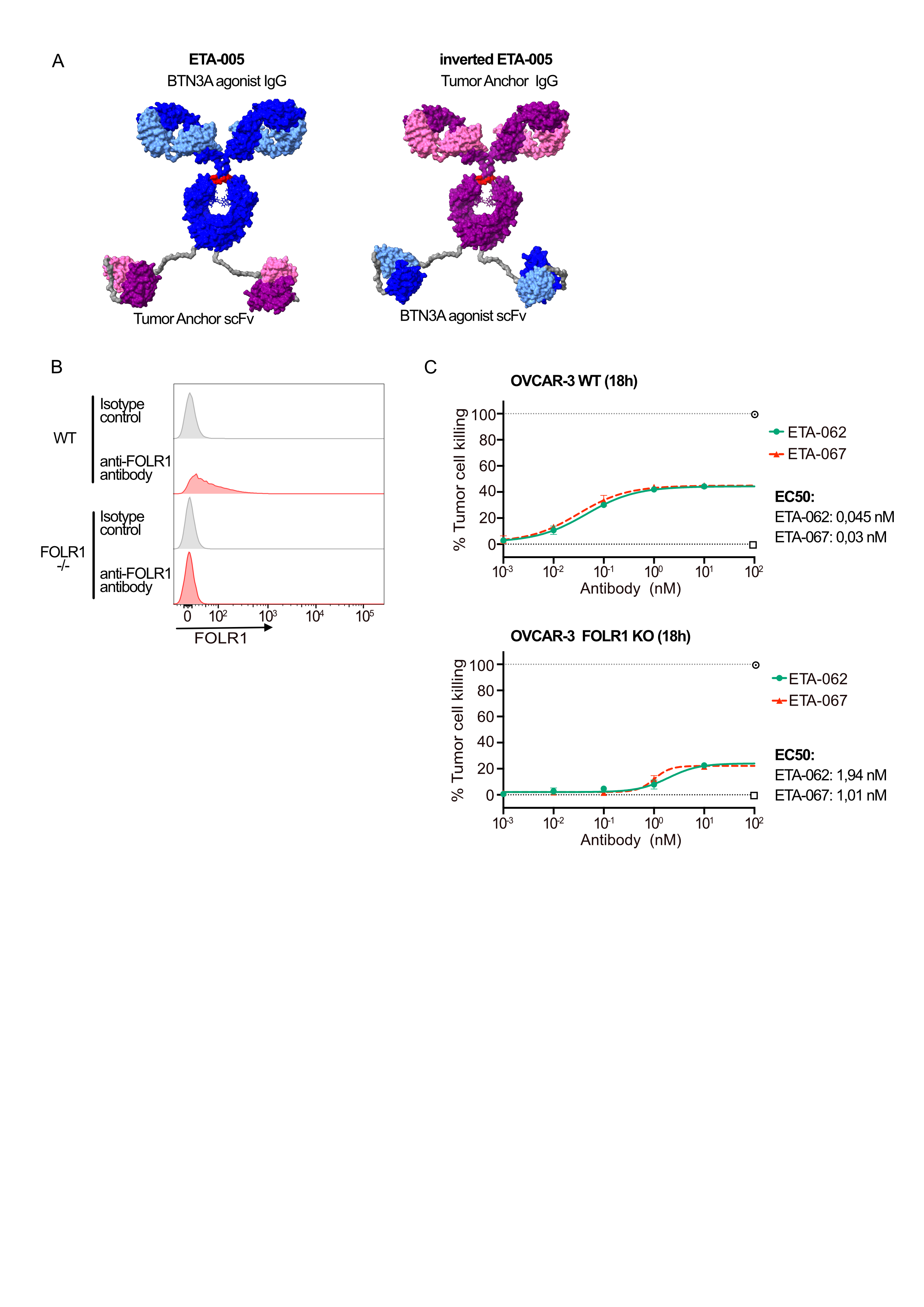


**Supplemental Figure 2: A:** Schematic representation of the overall structure of the classical antibody format, here ETA-005, in comparison with the inverted format with the tumor anchor binding V-regions in the IgG and BTN3A agonist binding V-regions in the scFv. **B:** Expression of FOLR1 in OVCAR-3 wild-type and FOLR1 knock-out cells (FOLR1 ­‑/‑). Grey histograms: incubation with isotype control; red histograms: incubation with anti-FOLR1 antibody. **C:** Comparison of the dose response curves and the calculated EC50-values for Evobodies ETA-062 and ETA-067. In a RTCA cytotoxicity assay, expanded Vγ9Vδ2 T cell lines from a healthy donor were co-cultured in triplicates with either OVCAR-3 wild-type (Top) or FOLR1 knock-out cells (bottom) and increasing concentrations of the respective Evobody for 16 h. Triton X-100 was added to determine maximum lysis and samples with medium alone served as negative control.


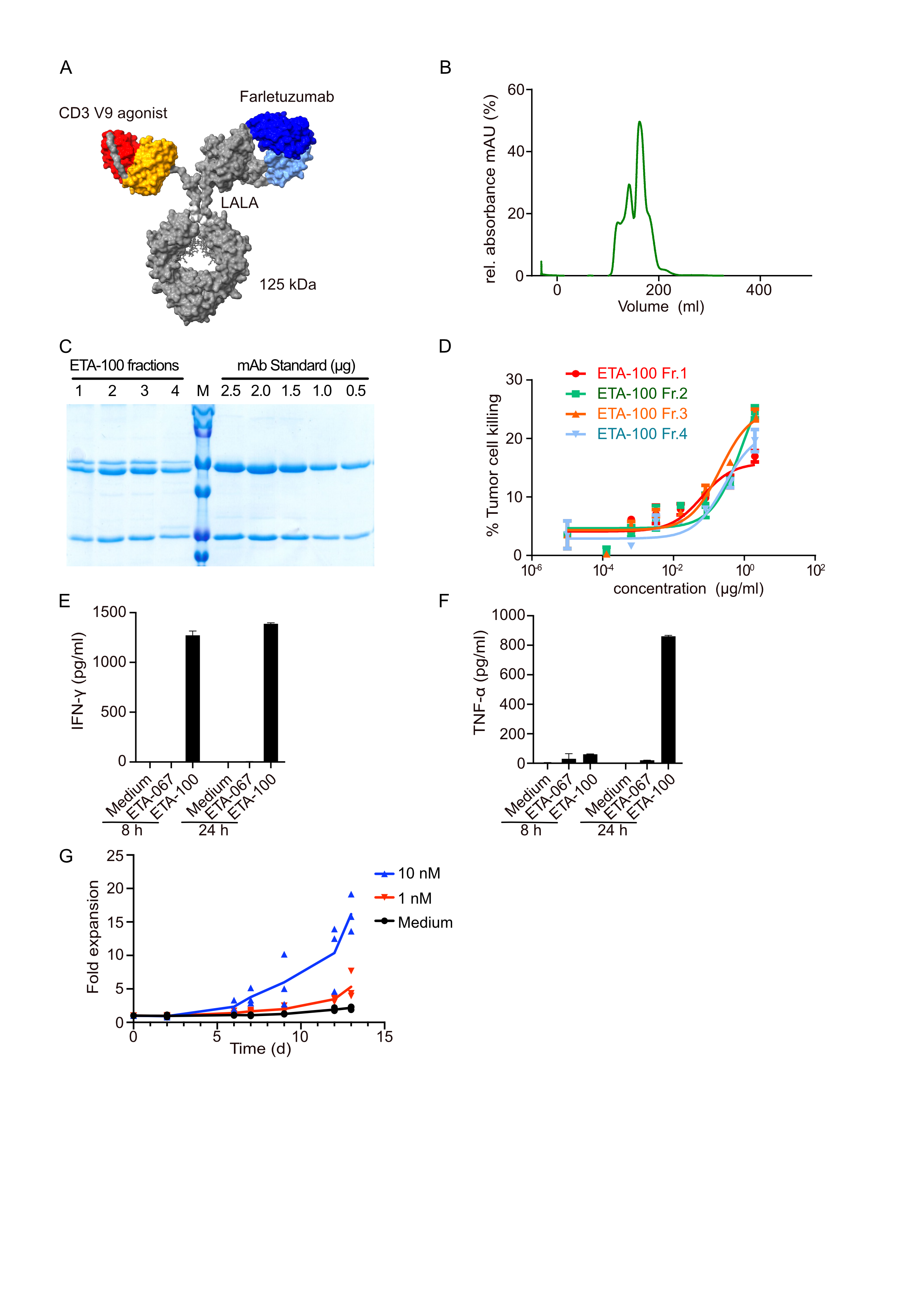


**Supplemental Figure 3: A-D:** Generation of the bispecific CD3 T cell engaging antibody ETA-100 for comparison with Evobodies. **A:** Schematic representation of antibody design. Red/yellow: CD3-binding scFv V9; Dark/light blue: variable Fragment of FOLR1-binding antibody Farletuzumab; Grey: antibody backbone. Total expected size is 125 kDa**. B:** Chromatogram of SEC of recombinant ETA-100. **C:** SDS-PAGE of the collected fractions from the SEC (1-4) in comparison to decreasing concentrations of therapeutic antibody rituximab. **D:** Comparison of dose response curves for the collected proteins from fractions 1 – 4 in a chromium release cytotoxicity assay with T cells from blood of healthy donors on FOLR1-positive SKOV-3 cells (two independent experiments with two different donors were performed in triplicates, representative shown). Fraction 3 was chosen for further experiments as it showed the highest efficiency. **E/F:** PBMCs from a healthy donor have been incubated in triplicates without OVCAR-3 target cells and 10 nM of the indicated bispecific antibodies ETA-067 and ETA-100. After 8 and 24 h the supernatant was taken off for quantification of IFN-γ **(E)** and TNF-α **(F)** via ELISA. **G:** Proliferation of Vδ2-negative T cells during the proliferation assay with healthy donors (n=3).
